# Supplementary material for: DNA-PKcs kinase activity stabilizes the transcription factor Egr1 in activated immune cells
Source: J Biol Chem. 2021 Sep 23;297(4):101209. doi: 10.1016/j.jbc.2021.101209 (PMC8551498; doi:10.1016/j.jbc.2021.101209)
Supplement: Figure S1 [file mmc1.pdf]

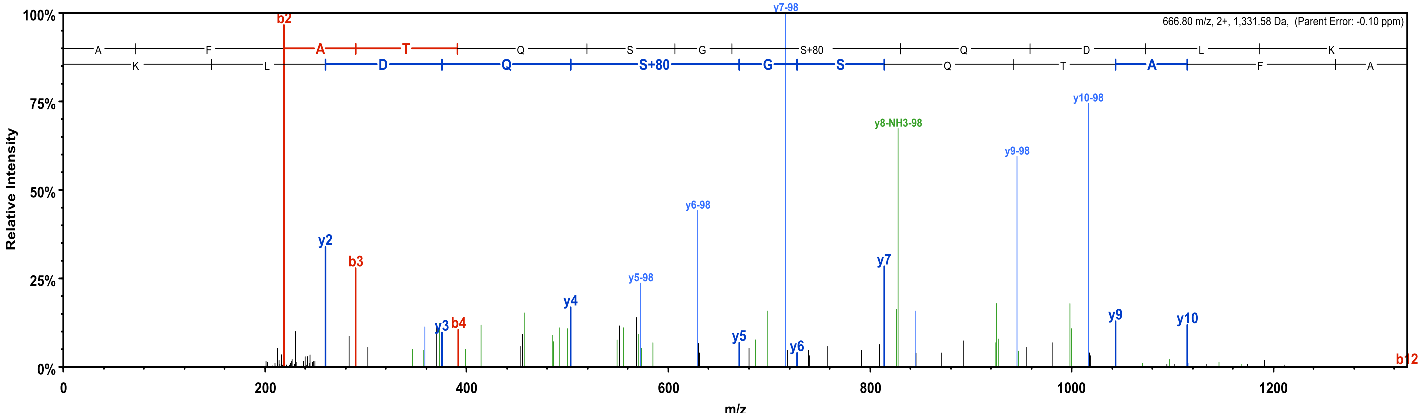


**Supporting Figure 1. Annotated spectrum of phosphorylated EGR1.** Sample spectrum for AFATSQGsQDLK tryptic peptide with a detectable Y ion series indicative of phosphorylation at S301 (the Y5 ion in this spectrum).
