# Supplementary material for: DNA-PKcs kinase activity stabilizes the transcription factor Egr1 in activated immune cells
Source: J Biol Chem. 2021 Sep 23;297(4):101209. doi: 10.1016/j.jbc.2021.101209 (PMC8551498; doi:10.1016/j.jbc.2021.101209)
Supplement: Figure S2 [file mmc2.pdf]

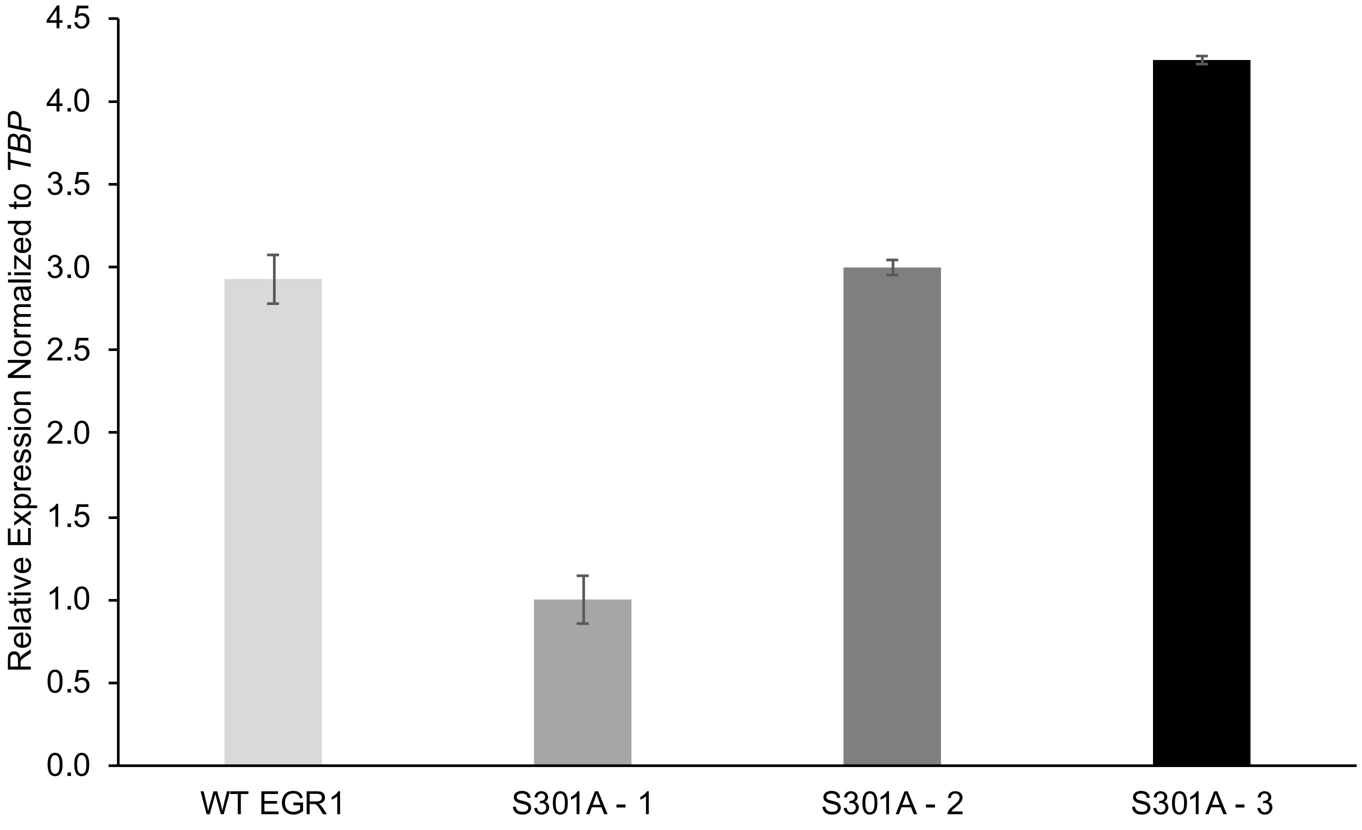

Supporting Figure 2: Egr1 S301 mRNA expression is similar to WT Egr1 mRNA expression. Real-time qPCR analysis of *EGR1* transcripts from CRISPR-generated S301A mutants. error bars = s.d. of the mean of technical replicates.
